# Examining the solutions to maintain psychological safety and nutritional and physical health of children during war: a review study

**Published:** 2019-08

**Authors:** Alizamen Salehifardjoneghani, Sedigheh Heydarisoreshjani, Ali Saljoghipeptani, Poran Khalafian

**Affiliations:** ^*a*^Development Management Shahrekord University of Medical Sciences, Shahrekord, Iran.; ^ran. *b*^Health Network of Responsible for Health Education in Koohrang City, Kohrang, Iran.; ^*c*^Head of Department of Family Health and Population Health, Deputy of Shahrekord University of Medical Sciences, Shahrekord, Iran.

## Abstract

**Background:**

War is a stressful factor for children and families. During war, there are two categories of children including those who are directly attacked and all of their amenities such as school, house, and social structure are influenced and destroyed. Another category includes children who are indirectly observing these disasters through social media and the first group, in addition to psychological aspects, suffers from nutritional and physical health problems. In this study, the researcher aims to find out if there is any solution to provide psychological safety and nutritional and physical health for children involved in war.

**Methods:**

In this study, articles published in English and Persian languages in scientific sites, journals and conferences of Medical Sciences of Iran and Red Crescent, SID, PubMed and Scopus, and humanities were searched by keywords such as psychological health safety, violence against children, vulnerability of children during war, nutritional and physical health of children, and solutions to provide supplies and health during war.

**Results:**

The results show that during war, it is necessary to gather information from the study area exposed to danger. Costs estimation, maintenance and storage of food supplies, supplying kitchens, warehouses and mobile refrigerators, providing food and shelter, establishment of hospitals and schools, education to cope with crisis for families and people working with children, and separating the consumer groups to maintain nutritional health of children are necessary. But the most important element is psychological health of children and it is necessary to train people before the accident and send them to the area. These people should know that children are more sensitive and understand the situations.

**Conclusions:**

To solve psychological problems of children, parents and authorities should prepare a comprehensive plan to reduce psychological injuries. For children who may need tranquilizers for psychological disorders, the presence of psychologist and psychiatrist is necessary. For health and nutrition of children, correct planning and prediction and management can reduce losses caused by the crisis.

**Keywords:**

Children, War, Psychological problems, Food supply, Health

